# Music performance anxiety from the challenge and threat perspective: psychophysiological and performance outcomes

**DOI:** 10.1186/s40359-020-00448-8

**Published:** 2020-08-25

**Authors:** Amélie J. A. A. Guyon, Regina K. Studer, Horst Hildebrandt, Antje Horsch, Urs M. Nater, Patrick Gomez

**Affiliations:** 1 Center for Primary Care and Public Health (Unisanté), University of Lausanne, Lausanne, Switzerland; 2 University of Applied Sciences and Arts Northwestern Switzerland, Olten, Switzerland; 3 Swiss University Center for Music Physiology, Basel University of the Arts, Basel, Switzerland; 4 Swiss University Center for Music Physiology, Zurich University of the Arts, Zurich, Switzerland; 5 Institute of Higher Education and Research in Healthcare (IUFRS), University of Lausanne, Lausanne, Switzerland; 6 Neonatology Service, Department Woman-Mother-Child, Lausanne University Hospital, Lausanne, Switzerland; 7 Department of Clinical & Health Psychology, University of Vienna, Vienna, Austria

**Keywords:** Music performance anxiety, Biopsychosocial model, Challenge, Threat, Respiration, Cardiovascular activity, Salivary cortisol, Salivary dehydroepiandrosterone, Salivary alpha-amylase, Music performance quality

## Abstract

**Background:**

Although many musicians perceive music performance anxiety (MPA) as a significant problem, studies about the psychobiological and performance-related concomitants of MPA are limited. Using the biopsychosocial model of challenge and threat as theoretical framework, we aim to investigate whether musicians’ changes in their psychobiological responses and performance quality from a private to a public performance are moderated by their general MPA level. According to the challenge and threat framework, individuals are in a threat state when the perceived demands of a performance situation outweigh the perceived resources, whereas they are in a challenge state when the perceived resources outweigh the perceived demands. The resources-demands differential (resources minus demands) and the cardiovascular challenge-threat index (sum of cardiac output and reverse scored total peripheral resistance) are the main indices of these states. We postulate that the relationship between general MPA level and performance quality is mediated by these challenge and threat measures.

**Methods:**

We will test 100 university music students reporting general MPA levels ranging from low to high. They will perform privately (i.e., without audience) and publicly (i.e., with an audience) on two separate days in counterbalanced order. During each performance session, we will record their cardiovascular and respiratory activity and collect saliva samples and self-reported measures. Measures of primary interest are self-reported anxiety, the resources-demands differential, the cardiovascular challenge-threat index, sigh rate, total respiratory variability, partial pressure of end-tidal carbon dioxide and the salivary biomarkers cortisol, dehydroepiandrosterone, and alpha-amylase. Both, the participants and anonymous experts will evaluate the performance quality from audio recordings.

**Discussion:**

The results of the planned project are expected to contribute to a more comprehensive understanding of the psychobiology of MPA and of the processes that influence musicians’ individual reactions to performance situations. We also anticipate the findings of this project to have important implications for the development and implementation of theory-based interventions aimed at managing musicians’ anxiety and improving performance quality. Thanks to the use of multimethod approaches incorporating psychobiology, it might be possible to better assess the progress and success of interventions and ultimately improve musicians’ chance to have a successful professional career.

**Trial registration:**

Not applicable.

## Background

Music performance anxiety (MPA) has been defined as « the experience of marked and persistent anxious apprehension related to musical performance (…), which is manifested through combinations of affective, cognitive, somatic and behavioral symptoms » [1] , p. 433. MPA is a significant problem for many musicians [2].

Performing publicly can be a psychophysiologically demanding activity for many musicians. Literature agrees that on average, state anxiety is higher before and during a public performance (i.e., in front of an audience or jury) compared to a private performance (i.e., without an audience or jury, e.g., [3, 4]). Compared to private performances, public performances are also characterized by enhanced physiological arousal in most musicians (e.g., [5, 6]). Research on the psychophysiological concomitants of MPA is scant. With regard to their subjective experience, general MPA level has been associated with significant increases in state anxiety from a private to a public performance [3, 7]. Regarding physiological measures, skin conductance, heart rate (HR), urinary adrenaline and noradrenaline did not show significant differences as a function of musicians’ general MPA level [3, 6, 8]. In contrast, analyses of the respiratory responses showed that the general MPA level significantly moderated changes from a private to a public performance in partial pressure of end-tidal carbon dioxide (P_et_CO_2_), total respiratory variability (quantified by the coefficient of variation) and sigh rate [7, 8]. Salivary cortisol (sC), salivary dehydroepiandrosterone (sDHEA), and salivary alpha-amylase (sAA) have yet to be considered in relation to potential MPA-associated differences in the context of music performance. The catabolic hormone cortisol and the anabolic hormone dehydroepiandrosterone (DHEA) are the main products of the hypothalamic-pituitary-adrenal (HPA) axis, a central regulatory system that in most healthy people is activated in response to psychosocial stressors, resulting in increased sC and sDHEA secretion (e.g., [9–12]. Anabolic balance is the ratio of DHEA to cortisol and has been suggested to be a sensitive indicator of well-being and health, more so than cortisol or DHEA alone [11, 13, 14]. Lower anabolic balance is associated with more unfavorable psychological well-being and health outcomes [14, 15]. SAA is an enzyme secreted from the salivary glands that has been increasingly used as a marker of the activity of the sympathoadrenal-medullary (SAM) axis, another important regulatory system involved in the response to psychosocial stressors [16–18]. Researchers have not investigated cardiovascular differences as a function of general MPA level other than for HR and HR variability (HRV) [8].

Not all musicians have the same predisposition when it comes to performing publicly; whereas some thrive, others fail [19, 20]. The general MPA level was shown to be a predictor of more negative self-rated performance among music students performing solo [21]. Few studies have evaluated whether musicians reporting relatively lower and higher general MPA levels differ in their expert-rated performance quality, and findings have been mixed [3, 6, 22]. No study has yet tested simultaneously whether differences in both self-rated and expert-rated performance quality between a private and a public performance vary significantly as a function of musicians’ general MPA level and what mechanisms might explain such effects.

One theoretical framework that offers a potential model to understand individual differences in psychophysiological responses and music performance quality (MPQ) as a function of musicians’ general MPA level is the biopsychosocial model (BPSM) of challenge and threat [23, 24]. This model provides a framework for understanding the motivational processes within the context of motivated performance situations. Motivated performance situations, such as test taking, athletic competitions, and music performances, are situations that require instrumental responses in order to attain valued self-relevant goals [23, 25]. According to the BPSM of challenge and threat, the self-relevance of the task-related goals drives task engagement [25]. Given task engagement, challenge and threat refer to two different states that a person can experience, depending on the balance between her/his subjective evaluations of demands and resources. Challenge occurs when evaluated resources meet or exceed evaluated demands, whereas threat arises when individuals evaluate demands as outweighing available resources. Challenge and threat are understood as labels representing anchors of a single bipolar continuum defined by the resources-demands differential, such that relative differences in challenge and threat (e.g., greater vs. lesser threat) are meaningful [26]. A central tenet of the BPSM of challenge and threat is that challenge and threat can be differentiated by means of specific cardiovascular patterns. The current view is that in the context of motivated performance situations, HR and ventricular contractility (defined as pre-ejection period (PEP) multiplied by − 1; PEP is the time in ms from the initiation of left ventricle contraction to aortic-valve opening) reflect task engagement proximally and goal relevance distally and are common across the challenge-threat continuum. Cardiac output (CO, the amount of liters of blood pumped by the heart per minute) and total peripheral resistance (TPR, an index of net constriction vs. dilation in the vascular system) differentiate challenge and threat. Relatively larger values of the cardiovascular challenge-threat index (CTI), defined as the sum of CO and of reverse scored TPR, reflect relatively greater challenge or lesser threat [24]. Compared to threat, during challenge, arteries are more dilated/less constricted, which facilitates the heart pumping relatively more blood. The cardiovascular pattern of challenge is supposed to be more conducive to approach-related goal pursuit, as blood delivery to the brain and muscles is more efficient [25, 26]. These indices have been extensively validated [23, 27, 28]. The cardiovascular patterns of challenge and threat have been associated with more positive and negative health outcomes, respectively [29–31].

Many studies have shown that individuals exhibiting a cardiovascular challenge pattern prior to cognitive and motor tasks perform better than individuals exhibiting a cardiovascular threat pattern (e.g., [32–35]). Assessing challenge and threat with cardiovascular measures, as opposed to self-reports only, confers advantages because this approach does not rely on individuals’ ability or willingness to accurately report on their experiences, particularly given that nonconscious and irrational influences are affecting them [36, 37]. Furthermore, cardiovascular challenge-threat indices better predict performance than self-reported variables [28, 32, 38, 39], thus further highlighting the usefulness of these physiological parameters. Most studies on the link between challenge/threat states and task performance have used a between-group design. A within-person design can more conclusively show that cardiovascular activity predicts performance quality by excluding the alternative hypothesis that individuals who possess greater ability at a task are more likely to react with a challenge state [28, 40]. Anxious pianists reported lower expectations of being able to complete several tasks relating to piano performance than non-anxious pianists [3], suggesting that increasing levels of MPA might be associated with greater threat/lesser challenge.

The aim of this study is to investigate in university music students (1) whether the differences in their psychophysiological responses and in their MPQ between a private performance and a public performance vary significantly as a function of their general MPA level and (2) whether the cardiovascular CTI and the resources-demands differential are significant mediators of the relationship between the general MPA level and the MPQ. We aim to test the following hypotheses:

1. *Relationship between general MPA level and challenge-threat*. Higher general MPA level is associated with greater increase in threat from the private to the public performance: With increasing general MPA level, change scores (public minus private) of the cardiovascular CTI and the resources-demands differential (self-rated resources minus self-rated demands) decrease.
2. *Relationship between general MPA level and MPQ.* Higher general MPA level is associated with greater decrease in self-rated and expert-rated MPQ from the private to the public performance: With increasing general MPA level, change scores (public minus private) of self-rated and expert-rated MPQ decrease.
3. *The mediating role of challenge-threat.* The cardiovascular CTI and the resources-demands differential are significant mediators of the relationships between general MPA level and self-rated and expert-rated MPQ.
4. *Relationship between general MPA level and state anxiety.* Higher general MPA level is associated with greater increase in state anxiety from the private to the public performance: With increasing general MPA level, change scores (public minus private) of state anxiety increase.
5. *Relationship between general MPA level and respiration.* Higher general MPA level is associated with greater increase in sigh rate and total respiratory variability and greater decrease in P_et_CO_2_: With increasing general MPA level, change scores (public minus private) of sigh rate and total respiratory variability increase, whereas change scores of P_et_CO_2_ decrease.
6. *Relationship between general MPA level and salivary measures of HPA axis activity and SAM axis activity.* Higher general MPA level is associated with greater increase in sC and greater decrease in sDHEA, anabolic balance and sAA: With increasing general MPA level, change scores (public minus private) of sC increase, whereas change scores of sDHEA, anabolic balance and sAA decrease.

## Methods/design

### Participants

Participants will be university music students recruited from university music schools by means of electronic invitations and posting on social media. Based on sample size estimations (see chapter Sample size calculation), we aim to collect complete data from 100 students.

Participants will have to be students in the department of classical music, between 18 and 35 years of age and in general good health. Exclusion criteria are cardiovascular, neurologic, respiratory and endocrine diseases, and the use of drugs with effects on the cardiovascular, nervous, respiratory and endocrine systems, including recreational drugs, beta-blockers and anxiolytic medication. Individuals wearing a pacemaker and working night shifts will also be excluded. A current diagnosis of panic disorder or eating disorders are additional exclusion criteria. Women will be excluded if they are pregnant or lactating. Eligibility to participate will be determined with an entry online questionnaire. Participants will receive 250 Swiss Francs as compensation if they complete all phases of the study protocol.

### Study protocol

**Fig. 1:**
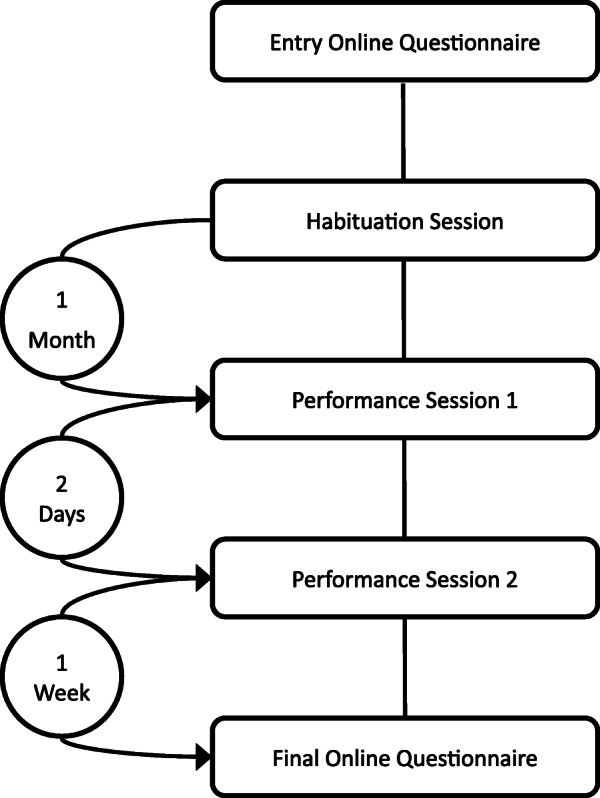
Study protocol

Participation in the study consists of completing an entry online questionnaire, attending three laboratory sessions (Habituation session, Performance session 1, Performance session 2) and completing a final online questionnaire (Fig. 1). The study is conducted in French or English depending on participants’ preference.

### Entry online questionnaire

In the entry online questionnaire, we will collect sociodemographic, academic, music and health-related data as well as the students’ general MPA level. Eligible participants will be contacted to arrange three appointments (Habituation session, Performance session 1, and Performance session 2). The two performance sessions will be scheduled ensuring that the participants have no other public performances neither on the same day of the performance session, nor the day before, or after it.

### Habituation session

Participants will be tested individually. Upon arrival to the lab, the experimenter will explain the study protocol to the participant and obtain written consent. This will be followed by the measurement of participant’s body height and weight, which are used to compute the body mass index (BMI). Afterwards, the participant will be familiarized with the physiological measurements. The experimenter will show the different instruments and explain their function. Afterwards, the sensors of the four devices Finometer, VU-AMS, BioRadio and Capnostream will be applied, and the participant will be asked to sit for 8 min alone. After removing the sensors of the Finometer and the Capnostream, the participant will be invited to play his/her instrument for a few minutes. Information about the comfort level regarding the sensors during the two periods will be obtained, and adjustments to the sensors will be made if required. The participant will also be familiarized with the saliva sampling procedure.

In the second part of the habituation session, the participant will be presented with a list of instrument specific music pieces from which he/she will have to choose one to perform during the following performance sessions. The pieces belong to the standard repertoire usually required for auditions, competitions and exams. The duration of the pieces is between 3 min and 4 min and 30 s (e.g., first 4 min of the solo parts of the clarinet concerto in A major, K. 622 by W.A. Mozart). We will provide the participants with the exact number of bars to perform and require them to perform the selected pieces by heart and without accompaniment during the two performance sessions. Participants will be given up to 2 days to choose their music piece. Finally, the experimenter will give the participant the following information about the upcoming performance sessions: the session order (private before public session or vice versa) and the audience composition (composed of the experimenter and five to seven music connoisseurs including two experts who will rate their MPQ). The experimenter will also explain the MPQ Scale to them.

### Private and public performance sessions

**Fig. 2:**
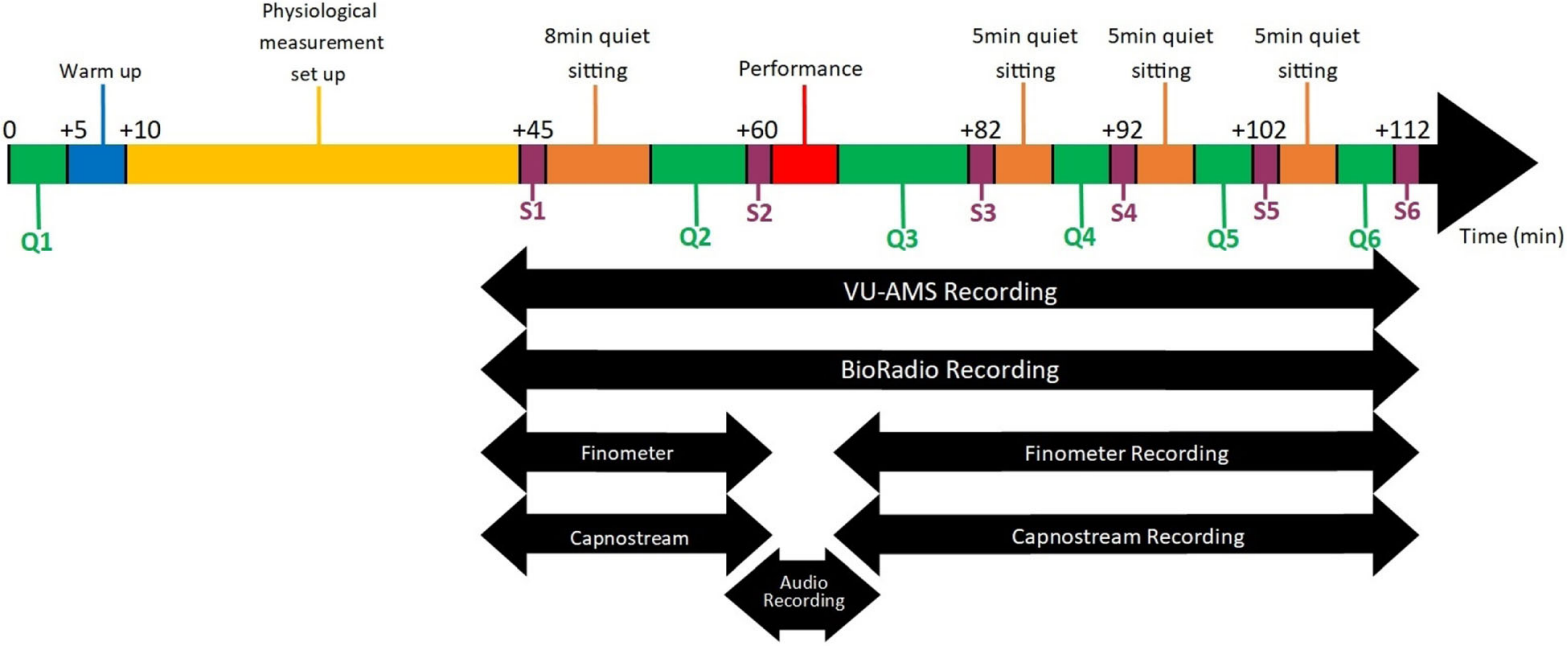
Performance session procedure – (Q = Questionnaire; S = Salivary sample) The quiet sitting periods correspond to a period where the participants are sitting alone at a table and required to keep their hands on the table, keep their eyes open, stay quiet and still and not cross their legs. Q1 consists of questions assessing participants’ compliance with the behavioral instructions (food intake, etc.) Q2 includes the questionnaires CSAI-2R (cognitive anxiety, somatic anxiety, self-confidence), self-reported demands and resources, STAI-6 and SAMq. Q3 includes the questionnaires FSS-2, MPQ Scale, CSAI-2R (somatic anxiety), self-reported demands and resources, STAI-6 and SAMq. Q4 and Q5 include the questionnaires CSAI-2R (somatic anxiety), STAI-6 and SAMq. Q6 includes the questionnaires CSAI-2R (somatic anxiety), STAI-6, SAMq, Post-Music Performance Thoughts Questionnaire, a question about performance engagement and a question about practice time.

One month after the habituation session, the participant will come to our laboratory for the two performance sessions. For each participant, the two sessions will take place 2 days apart (e.g., Monday and Wednesday) at the same time of the day. Participants will be scheduled at either 1 pm or 3.45 pm. The procedures of the two sessions are identical, except for the fact that the participants will perform without audience in the private session and in front of an audience of six to eight persons in the public session. Participants are randomly assigned to one of the two possible orders. The procedure of a performance session is shown in Fig. 2.

The day before each performance session, the participants will receive an electronic reminder in which they are asked to comply with the following requirements: no alcohol intake and no intense physical activity 24 h before the visit, no heavy meal 1h15min before the visit, no caffeine intake (including coffee, tea or chocolate) 1h15min before the visit, no cigarettes or any products containing nicotine 1 h before the visit, and no food intake 15 min before the visit. The email for the public performance session will also remind the participants of the audience composition.

First, participants will be given 5 min to prepare their instrument and warm up. Then, they will fill in a first questionnaire (Q1) to verify their compliance with the behavioral instructions (food intake, etc.). After attaching the sensors of the Finometer, VU-AMS, BioRadio and Capnostream and performing the required checks and calibrations, a first saliva sample (S1) will be collected followed by an 8-min period during which the participants will be sitting alone at a table and required to keep their hands on the table, keep their eyes open, stay quiet and still and not cross their legs. Afterwards, participants will fill in a second questionnaire (Q2) and take a second saliva sample (S2). Following the removal of the sensors of the Finometer and of the Capnostream, the participants will perform their music piece. After the performance, the participants will fill in a third questionnaire (Q3) and take a third saliva sample (S3). The rest of the session will consist of three 5-min sitting periods each followed by questionnaires (Q4, Q5, Q6) and saliva collection (S4, S5, S6). The timing of the sitting periods, performance, questionnaires, and saliva collection, as well as the content of each questionnaire are given in Fig. 2.

Performances will be recorded with a handy recorder Zoom H4 (Zoom North America, Hauppauge, NY, USA) placed on a tripod close to the participant. It will be combined with a microphone DPA 4099 (DPA Microphones, Inc., Longmont, CO, USA), which will be placed on the participant’s instrument with a clip or on a music stand depending on participant’s preferences (only on a music stand for singers). Audio recording will be started and stopped by the experimenter while participants are completing questionnaire Q2 and Q3, respectively.

### Final online questionnaire

One week after completing the second performance session, participants will receive the recordings of their two performances via an e-mail containing a link to a final online questionnaire and a Dropbox link to two mp3 audio files named “Recording1” for the first performance session and “Recording2” for the second performance session. In the online questionnaire, participants will be asked to listen to these audio files in the same order and rate the MPQ using the MPQ Scale.

Finally, the participant will fill in questionnaires assessing their trait anxiety, social anxiety and depressive symptoms, all of which are potential confounding variables because they can affect the psychophysiological measures.

### Music performance quality evaluated by anonymous experts

Upon termination of the experiments with all participants, the audio recordings will be sent to music experts for evaluation of the performances using the MPQ Scale. The experts will be professional musicians/teachers with a minimum requirement of master’s level training on their main performance instrument and with experience of professional assessment and adjudication (see [41–43] for similar criteria). They will have no association with the participants. We will have five judges per instrument type [44, 45]. In order to minimize the influence of nonmusical factors on the evaluations [46, 47], judges will receive the recordings in an individually randomized order, work independently and be unaware of the conditions under which performances were recorded and of any personal data about the participants.

### Measures of the online entry questionnaire

#### Sociodemographic data

The sociodemographic data include age, gender, mother tongue, French or English level (on a scale from 1 = Do not speak and do not understand English/French to 5 = Speak and understand perfectly English/French), and night shift work (yes/no).

#### Academic and music-related data

The academic and music-related data include the name of the school, the department, the current academic year, the main instrument, the time they started to play their main instrument, the second instrument, the average number of hours per day of music practice, the number of public solo and ensemble performances given during the past 12 months.

#### Health-related data

We will ask participants to list any known disease and any acute or chronic medication intake and to answer the questions assessing panic disorder and eating disorders from the Patient Health Questionnaire (for English version [48]; for French version [49]). Women will be also asked to indicate whether they are pregnant, lactating or using hormonal contraceptives. Women will also indicate the first day and the length of their last period as well as the duration of their menstrual cycle. Moreover, participants will have to indicate if they wear a pacemaker, smoke or take recreational drugs.

#### General MPA level

The general MPA level will be assessed with the state scale of the State-Trait Anxiety Inventory (STAI-S; for English version [50], for French version [51]), which consists of 20 items, e.g., “I am tense,” rated on a 4-point Likert scale (1 “not at all” to 4 “very much so”). The score ranges from 20 (no anxiety) to 80 (severe anxiety). Because anxiety depends on the performance setting [52], we will ask students to indicate how they generally feel when they perform solo. We [8, 21] and others [e.g., [53, 54, 55]] have used this instrument to assess the general MPA level. The internal consistency of this questionnaire is excellent (Cronbach’s alpha > .90 [8, 21]).

The Kenny Music Performance Anxiety Inventory-Revised (for English version [56], for French version [57]) will be used as a complementary measure to the STAI-S. This questionnaire consists of 40 items evaluating eight MPA-related dimensions (proximal somatic anxiety and worry about performance, worry/dread focused on self/other scrutiny, depression/hopelessness, parental empathy, memory, generational transmission of anxiety, anxious apprehension and biological vulnerability). Each item is rated on a 7-point Likert scale (0 “Strongly disagree” to 6 “Strongly Agree”). The score can vary from 0 (no anxiety) to 240 (severe anxiety). The psychometric properties of this questionnaire are good [58].

### Performance session measures

For the measures assessed during the two performance sessions, we distinguish between primary and secondary measures. Primary measures are relevant to the study hypotheses. Secondary measures are assessed and analyzed for exploratory purposes.

#### Cardiovascular measures

Cardiovascular measures will be recorded and analyzed following international guidelines [59–61]. The VU-AMS (Free University, Amsterdam, The Netherlands) will be used to obtain impedance cardiographic and electrocardiographic recordings by applying seven electrodes on specific positions of participants’ thorax and back. We will analyze the data with the VU-AMS Data, Analysis & Management Software (Free University, Amsterdam, The Netherlands). The Finometer (FMS Finapres Medical Systems, Amsterdam, The Netherlands) will be used to record blood pressure (BP) beat-to-beat by finger-cuff photoplethysmography. A finger cuff is wrapped around the middle phalanx of the middle finger of participants’ left hand. A hydrostatic height correction of the finger with respect to the heart level is active during the entire recording. The Finometer recordings will be analyzed with the Beatscope software (FMS Finapres Medical Systems, Amsterdam, The Netherlands). Beat-to-beat BP recording is a significant advancement from the more often used single BP readings. The finger cuff will not be used during the performance, as it would interfere with performing. The primary cardiovascular measure is the CTI defined as the sum of CO and reverse scored TPR [33]. HR, HRV, stroke volume, systolic BP, diastolic BP, mean arterial pressure, CO, TPR and PEP are secondary cardiovascular measures.

#### Respiratory measures

Respiratory inductive plethysmography (RIP) sensors of the BioRadio (Great Lakes NeuroTechnologies, OH, USA) will be used to measure breathing parameters. This system consists of a thoracic band and an abdominal band that provide thoracic and abdominal motion signals, respectively. The proportionality constant between the thoracic and abdominal motion signals is determined by the qualitative diagnostic calibration method [62]. The RIP bands will be volume-calibrated using a spirometer (Vernier Software & Technology, Beaverton, OR, USA) at the beginning of each recording. The BioRadio recordings will be analyzed with the Vivosense software (Vivonoetics, Newport Beach, CA, USA). P_et_CO_2_ will be recorded by means of a nasal cannula connected to a nondispersive infrared CO_2_ monitor (Capnostream 35, Medtronic, Switzerland). The nasal cannula will not be used during the performance, as it would interfere with performing. The primary respiratory measures are sigh rate, total respiratory variability and P_et_CO_2_. Secondary respiratory measures include means of inspiratory time, expiratory time, total breath duration, tidal volume and minute ventilation. An embedded motion sensor in the BioRadio will record triaxial acceleration and angular velocity to quantify movement, both of which could affect other physiological measures.

#### Salivary measures

Saliva samples will be obtained via a passive drooling method facilitated by polypropylene straws into low-bind polypropylene 2 mL cryovials (Salicap, IBL International, Hamburg, Germany). For each saliva sample, participants will be instructed to rinse their mouth with water, swallow the saliva currently in their mouth, accumulate new saliva for 2 min and then transfer all saliva into the tubes. Samples will be stored immediately after collection at − 20 °C and then shipped on dry ice to the Biochemical Laboratory of the Department of Clinical Psychology, University of Vienna headed by Prof. U.M. Nater, where they will be assayed for free cortisol and DHEA using immunoassay kits and for sAA using enzyme kinetic assay kits. SC, sDHEA and sAA are primary measures.

#### Self-reported demands and resources

Self-reported demands will be assessed before the performance (Q2) with the question “How demanding do you expect this music performance to be?” and after the performance (Q3) with the question “How demanding was the music performance situation?”. Self-reported resources will be assessed before the performance (Q2) with the question “How able are you to cope with the demands of the music performance?” and after the performance (Q3) with the question “How able were you to cope with the demands of the music performance situation?”. The participants will answer using a 6-point Likert scale ranging from 1 “not at all” to 6 “extremely”. These questions are adapted from the cognitive appraisal ratio [63, 64]. The resources-demands differential is the primary measure obtained by subtracting the score of the first question from the score of the second question. The self-reported demands and self-reported resources analyzed separately are secondary measures.

#### State anxiety

Self-rated state anxiety is a primary measure assessed before and after each performance (Q3 to Q6) with the 6-item version of the STAI (STAI-6; example item: “I am tense”), which yields results that are comparable to those obtained using the full-form [65]. Each item is rated on 4-point Likert scale (1 “not at all” to 4 “very much so”) and a sum score of all items is computed. Higher scores indicate greater state anxiety.

#### Music performance quality

MPQ is a primary measure assessed after each performance (Q3) with the MPQ Scale (See Additional file 1). This scale is a revised version of a MPQ scale used in a previous study [21]. The scale consists of nine dimensions (“tempo”, “rhythm”, “intonation”, “tone”, “dynamics”, “articulation”, “musical understanding and interpretation”, “missing notes, wrong notes and unwritten breaks” and “global appreciation”) to be rated on a 21-point scale ranging from 1 (= lowest score) to 6 (= highest score), with 0.25-point intervals to replicate the grading system of French-speaking Swiss schools. A definition is given for each dimension together with the specific aspects to consider in scoring the dimension. As done previously [21], we will compute an average score of these dimensions with higher scores corresponding to better MPQ.

#### Cognitive anxiety, somatic anxiety and self-confidence

Cognitive anxiety, somatic anxiety, and self-confidence are secondary measures. Cognitive anxiety and self-confidence will be assessed before each performance (Q2), whereas somatic anxiety will be assessed before and after each performance (Q2 to Q6) with the Competitive State Anxiety Inventory – 2 Revised (CSAI-2R; for English [66], for French [67]). Five items (e.g., “I am concerned about performing poorly”) assess cognitive anxiety, seven items (e.g., “My heart is racing”) assess somatic anxiety and five items (e.g., “I’m confident of coming through under pressure”) assess self-confidence. Intensity of each item is rated on a 4-point Likert scale (1 = “not at all” to 4 = “very much so”). We have changed the instructions to make the inventory applicable to music performance and the internal consistency of this questionnaire is good (Cronbach’s alpha > .80 [68]).

#### Arousal, valence, control

The affective dimensions arousal, valence, and control are secondary measures assessed with the Self-Assessment Manikin (SAMq; for English version [69], for French version [70]) before and after each performance (Q2 to Q6). The SAMq consists of five manikins for each affective dimension. Each dimension is scored on a 5-point scale. Arousal ranges from 1 (low arousal) to 5 (high arousal). Valence ranges from 1 (negative valence) to 5 (positive valence). Control ranges from 1 (not in control) to 5 (in control).

#### Flow

Flow refers to the subjective experience of being immersed and absorbed in an activity and is suggested to be a state of consciousness that is conducive to high levels of performance. In this study, it is a secondary measure assessed after each performance (Q3) with the Flow State Scale-2 (FSS-2) (for English [71], for French [72]). It consists of 36 items, with four items for each of nine dimensions (“challenge-skill balance”, “merging of action and awareness”, “clear goals”, “unambiguous feedback”, “concentration on the task at hand”, “sense of control”, “loss of self-consciousness”, “transformation of time” and “autotelic experience”). Respondents indicate the extent to which they agree with each statement on a 5-point Likert scale from 1 “strongly disagree” to 5 “strongly agree” by referring to the just completed performance. Higher scores reflect more intense flow. The internal consistency of this questionnaire is good (Cronbach’s alpha >.80 [71]).

#### Negative and positive post-music performance thoughts

Negative and positive post-music performance thoughts are secondary measures assessed after each performance (Q6) with the 23-item Post-Music Performance Thoughts Questionnaire [21] adapted from the Post-Event Rumination Questionnaire for public speech [73]. The questionnaire is divided into nine positive items (e.g., “My concert was good”) and 14 negative items (e.g., “I made a lot of mistakes”). Participants will be instructed to report to what extent they had each thought on mind since the end of the performance using a 5-point Likert scale ranging from 0 “not at all” to 4 “extremely”. The positive items score varies from 0 (no positive thoughts) to 36 (a lot of positive thoughts), and the negative items score varies from 0 (no negative thoughts) to 56 (a lot of negative thoughts). Internal consistencies of both scales are excellent [21].

#### Task engagement

The engagement in the task is a secondary measure assessed at the end of each performance session (Q6) with the question “To what extent did you try to give the best of yourself during the performance?”. Participants will answer using a 5-point Likert scale ranging from 1 “not at all” to 5 “very much so”.

#### Practice time

The amount of practice time for each session performance is a secondary measure assessed at the end of each performance session (Q6) with the question “How much time have you spent in the last 48 hours specifically preparing the musical piece you have just played?”. Participants will respond by indicating a numerical value.

#### Measures of the final online questionnaire

##### *Trait anxiety*

Trait anxiety will be assessed with the trait scale of the State-Trait Anxiety Inventory for adults (for English version [50], for French version [51]. This questionnaire consists of 20 items (example item: “I am a steady person”) that are rated on a 4-point Likert scale (1 “almost never” to 4 “almost always”). The total score ranges from 20 to 80, with higher scores indicating greater trait anxiety. The test has good psychometric properties [50, 74].

##### *Social anxiety*

Social anxiety will be assessed with the self-reported version of the Liebowitz Social Anxiety Scale (for English version [75, 76], for French version [77]). This scale quantifies fear and avoidance of social interaction situations (12 items, e.g., “Calling someone you do not know very well”) and performance situations (12 items, e.g., “Acting, performing or giving a talk in front of an audience”) during the last week. The possible responses for experienced fear are 0 (none), 1 (mild), 2 (moderate), or 3 (severe), and the possible responses for avoidance behavior are 0 (never), 1 (occasionally), 2 (often), or 3 (usually). The total score can range from 0 to 144 with higher scores indicating greater social anxiety. The test possesses good psychometric properties [78]*.*

##### *Depressive symptoms*

Depressive symptoms will be assessed with the Beck Depression Inventory-II (for English version [79], for French version [80]). This inventory is a unipolar questionnaire assessing depressive symptoms during the last 2 weeks with 21 items. Each item contains four sentences, which are coded from 0 (less close to depression, e.g., “I do not feel sad”) to 3 (*closest to depression*, e.g., “I am so sad or unhappy that I can’t stand it”). The total score can range from 0 to 63 with higher scores indicating more severe depressive symptoms. The test has good psychometric properties [79].

### Data-analytic strategy

Scores for self-report measures will be computed in accordance with guidelines. For cardiovascular and respiratory measures, scores will be computed for the 8-min sitting period, the 4-min performance period, and the three 5-min post-performance sitting periods, with focus on the 8-min sitting period, as this is the most relevant period with regard to our hypotheses. The area under the curve with respect to ground and to increase will be calculated for the salivary parameters [81].

Given the repeated measures design, we will use multilevel mixed-effects modeling following state of the art procedures [82, 83]. For the mediation analyses, we will follow the model suggested by Baron and Kenny [84]. The main predictors are performance session (private vs. public), general MPA level and their interaction. Where appropriate, skewed variables will be transformed. We will use an alpha level of 0.05 for all tests. We will perform sensitivity analyses adding control variables to the models if they are theoretically meaningful and significantly related to the outcome variable. Potential control variables are age, gender, use of hormonal contraceptives, menstrual cycle phase, BMI, smoking, trait anxiety, social anxiety, depressive symptoms, academic year, practice time, session order, and motion.

### Sample size calculation

The sample size calculation refers to the first three sets of hypotheses, which we consider to be the critical ones. The power computations are based on a model by which the effect of general MPA level on MPQ follows two paths, direct and indirect. In the indirect path, MPA acts on the cardiovascular CTI, which acts on MPQ. The computations rely on the repeated simulations of such a model. First, we fix the distributional parameters of MPA. We obtained a mean (*M*) of 45 on the STAI-S scale and a standard deviation (*SD)* of 12 [8]. We assume that general MPA level influences deltaCTI, i.e., the difference in CTI between private and public performance sessions. To simulate realistic values for deltaCTI, we rely on [85] who reported a difference in the cardiovascular CTI between a “challenge” group and a “threat” group of 0.9. Thus, we assume *M* = 0.9 for deltaCTI. Moore et al. [85] used a two-group design, while we use a repeated-measures design. To estimate *SD* of deltaCTI, we rely on [8] who used a similar design as in the planned project. In their study, *M* and *SD* of deltaHR between private and public sessions were 28 and 16, respectively. Assuming similar coefficients of variation for deltaHR and deltaCTI, we obtain *SD* = 0.9*16/28 = 0.5 for deltaCTI. From knowledge about distributions of general MPA level and deltaCTI and assuming normal distributions and linear relationships, we can deduce the equation of the linear regression from the following variance components: Var(deltaCTI) = variance of deltaCTI, Var_Res_ = residual variance, Var(MPA) = variance of MPA. The slope beta_deltaCTI_MPA_ is the square root of (Var(deltaCTI) – Var_Res_)/Var(MPA), and the intercept is mean(deltaCTI) - beta_deltaCTI_MPA_*mean(MPA). Defining the effect size using Cohen’s [86] η^2^, where η^2^ = (Var(deltaCTI)-VarRes)/ Var(deltaCTI), the model relating MPA and deltaCTI is specified. Next, we specify the effect of deltaCTI on deltaMPQ, where deltaMPQ is the difference in MPQ between public and private performance sessions. We assume a linear relationship with slope beta_deltaMPQ_deltaCTI_. Without any loss of generality, we can specify the linear relation without intercept and with residual *SD* = 1. Thus, the indirect effect of MPA on deltaMPQ is beta_indir_ = beta_deltaCTI_MPA_*beta_deltaMPQ_deltaCTI._ Next, we assume that there is also a direct effect given by the slope beta_dir_, which is determined by the assumed mediated proportion (MP): beta_dir_ = beta_deltaCTI_MPA_*beta_deltaMPQ_deltaCTI_*(1-MP)/MP. In summary, given a simulation of MPA, we simulate deltaCTI and deltaMPQ = beta_deltaMPQ_deltaCTI_ *deltaCTI + beta_dir_*MPA + e, e ~ N(0,1). Finally, we define that small-to-medium effects (according to [86] criteria for interpreting η^2^) of MPA on deltaMPQ, MPA on deltaCTI and deltaCTI on deltaMPQ and MP between 20 and 30% are scientifically meaningful. By running 10′000 simulations of the aforementioned model with different combinations of the parameters, we determine that these effects are significant at α = 0.05 and power > 0.80 with a sample of 100 participants.

## Discussion

The findings of this study will allow us to determine to what extent MPA can be examined and understood from the perspective of the BPSM of challenge and threat and to what extent the BPSM of challenge and threat is a valid and useful framework by which performance variability can be examined, understood, and predicted in the domain of music performance. Furthermore, by investigating both psychophysiological and performance-related concomitants of a form of anxiety that has so far received relatively little attention, this study will add new and important information that will contribute to the issue of generalizability of findings across subtypes of social anxiety (disorder) and anxiety (disorders) more broadly. We also anticipate the outcomes of this study to contribute significantly to guiding the development and implementation of theory-based interventions aimed at managing musicians’ anxiety and improving performance quality. Psychophysiological monitoring adds an important dimension to the diagnostic and intervention outcome assessments. Thanks to the use of multimethod approaches incorporating psychobiology, it might be possible to better assess the progress and success of interventions and ultimately improve musicians’ chance to have a successful career. Within these approaches, respiratory regulation may play a significant role. Breathing is a powerful regulator of homeostatic balance and can be controlled voluntarily making breathing modification an accessible intervention goal. Breathing modification might be an integral part of interventions aimed at promoting challenge states over threat states. Given that the cardiovascular response pattern characterizing a threat state is considered to have deleterious health consequences when experienced frequently, such interventions may also have health-related implications. Finally, motivated performance situations are pervasive, in particular in the workplace. Thus, we expect the results of this study to have implications that extend beyond the domain of music performance.

### Possible challenges

We are conscious that this project is ambitious and that we might face several challenges. We discuss three of them. A first challenge would be having a similar number of male and female participants to avoid a gender bias. Previous studies on the same population [7, 21] showed that, on average, 60 to 67% of the sample were female. Having a well-balanced gender ratio is important considering gender differences in the prevalence of anxiety disorders [87, 88] and especially of MPA [2], and in the psychophysiological response to stressors [89]. The second challenge that we might face is related to women’s menstrual cycle phase. The monthly fluctuation in the levels of steroid hormones in women affect their psychophysiology (e.g., [90–93]). Following previous research [14, 94], we will control for these effects by testing female participants during the first 7 days after menstruation (follicular phase). To achieve this, we will collect information about the first day of the last menstrual cycle, the typical duration of the period, and of the entire cycle. We will use this information to schedule the appointments for the performance sessions. However, women’s menstrual cycle is not always regular, making it hard to correctly predict the right period to book an appointment, especially one or 2 months in advance. A final challenge we might face relates to any obstacles that could interfere with the smooth completion of the study such as malfunctioning devices or a high dropout rate of participants. In these cases, we will recruit new participants in order to achieve a total sample size of 100 participants with complete data, in accordance with the sample size estimation.

## Supplementary information

**Additional file 1.** Music performance Quality (MPQ) Scale. This scale is used to evaluate the quality of the music performances.

## Data Availability

Not applicable.

## Abbreviations

BMI: Body mass index
BP: Blood pressure
BPSM: Biopsychosocial model
CO: Cardiac output
CSAI-2R: Competitive state anxiety inventory - 2 revised
CTI: Cardiovascular challenge-threat index
DHEA: Dehydroepiandrosterone
FSS-2: Flow state scale-2
HPA: Hypothalamic-pituitary-adrenal
HR: Heart rate
HRV: Heart rate variability
MP: Mediated proportion
MPA: Music performance anxiety
MPQ: Music performance quality
PEP: Pre-ejection period
P_et_CO_2_: Partial pressure of end-tidal carbon dioxide
Q1 to Q6: Questionnaire 1 to 6
RIP: Respiratory inductive plethysmography
S1 to S6: Saliva sample 1 to 6
sAA: Salivary alpha-amylase
SAM: sympathoadrenal-medullary
SAMq: Self-assessment manikin
sC: Salivary cortisol
sDHEA: Salivary dehydroepiandrosterone
STAI-6: Short version of the State-Trait Anxiety Inventory
STAI-S: Adaptation of the state scale of State-Trait Anxiety Inventory to performance setting
TPR: Total peripheral resistance

## References

[1] Kenny DT, Juslin PN, Sloboda J (2010). The role of negative emotions in performance anxiety. Handbook of music and emotion: theory, research, applications.

[2] Fernholz I, Mumm JLM, Plag J, Noeres K, Rotter G, Willich SN, Schmidt A (2019). Performance anxiety in professional musicians: a systematic review on prevalence, risk factors and clinical treatment effects. Psychol Med.

[3] Craske MG, Craig KD (1984). Musical performance anxiety: the three-systems model and self-efficacy theory. Behav Res Ther.

[4] Fancourt D, Aufegger L, Williamon A (2015). Low-stress and high-stress singing have contrasting effects on glucocorticoid response. Front Psychol.

[5] Aufegger L, Wasley D. Salivary cortisol and alpha-amylase are modulated by the time and context of musical performance. Int J Stress Manag. 2018;25(S1):81–93.

[6] Fredrikson M, Gunnarsson R (1992). Psychobiology of stage fright: the effect of public performance on neuroendocrine, cardiovascular and subjective reactions. Biol Psychol.

[7] Guyon AJAA, Cannavò R, Studer RK, Hildebrandt H, Danuser B, Vlemincx E, Gomez P. Respiratory variability, sighing, anxiety, and breathing symptoms in low-and high-anxious music students before and after performing. Front Psychol. 2020;11:303. 10.3389/fpsyg.2020.00303 PMC7054282 32174869

[8] Studer RK, Danuser B, Hildebrandt H, Arial M, Wild P, Gomez P (2012). Hyperventilation in anticipatory music performance anxiety. Psychosom Med.

[9] Dickerson SS, Kemeny ME (2004). Acute stressors and cortisol responses: a theoretical integration and synthesis of laboratory research. Psychol Bull.

[10] Kaltsas GA, Chrousos GP, Cacioppo JT, Tassinary LG, Berntson GG (2007). The neuroendocrinology of stress. Handbook of psychophysiology.

[11] Lam JC, Shields GS, Trainor BC, Slavich GM, Yonelinas AP (2019). Greater lifetime stress exposure predicts blunted cortisol but heightened DHEA responses to acute stress. Stress Health.

[12] Lennartsson AK, Kushnir MM, Bergquist J, Jonsdottir IH (2012). DHEA and DHEA-S response to acute psychosocial stress in healthy men and women. Biol Psychol.

[13] Epel ES, McEwen BS, Ickovics JR (1998). Embodying psychological thriving: physical thriving in response to stress. J Soc Issues.

[14] Mendes WB, Gray HM, Mendoza-Denton R, Major B, Epel ES (2007). Why egalitarianism might be good for your health: physiological thriving during stressful intergroup encounters. Psychol Sci.

[15] Rasmusson AM, Vasek J, Lipschitz DS, Vojvoda D, Mustone ME, Shi Q, Gudmundsen G, Morgan CA, Wolfe J, Charney DS (2004). An increased capacity for adrenal DHEA release is associated with decreased avoidance and negative mood symptoms in women with PTSD. Neuropsychopharmacology..

[16] Ali N, Nater UM. Salivary alpha-amylase as a biomarker of stress in behavioral medicine. Int J Behav Med. 2020;27:337–42. 10.1007/s12529-019-09843-x PMC7250801 31900867

[17] Thoma MV, Kirschbaum C, Wolf JM, Rohleder N (2012). Acute stress responses in salivary alpha-amylase predict increases of plasma norepinephrine. Biol Psychol.

[18] Warren CM, van den Brink RL, Nieuwenhuis SB, Jos A (2017). Norepinephrine transporter blocker atomoxetine increases salivary alpha amylase. Psychoneuroendocrinology.

[19] Studer RK, Danuser B, Wild P, Hildebrandt H, Gomez P (2014). Psychophysiological activation during preparation, performance, and recovery in high- and low-anxious music students. Appl Psychophysiol Biofeedback.

[20] Yoshie M, Kudo K, Ohtsuki T (2008). Effects of psychological stress on state anxiety, electromyographic activity, and arpeggio performance in pianists. Med Probl Perform Artists.

[21] Nielsen C, Studer RK, Hildebrandt H, Nater UM, Wild P, Danuser B, Gomez P (2018). The relationship between music performance anxiety, subjective performance quality and post-event rumination among music students. Psychol Music.

[22] Kusserow M, Candia V, Amft O, Hildebrandt H, Folkers G, Tröster G. Monitoring stage fright outside the laboratory: an example in professional musician using wearable sensors. Med Probl Perform Artists. 2012;27(1):21–30. 22543319

[23] Blascovich J. Challenge and threat. In: Elliot AJ, editor. Handbook of approach and avoidance motivation. New York, NY: Psychology Press; 2008. p. 431–45.

[24] Seery MD (2011). Challenge or threat? Cardiovascular indexes of resilience and vulnerability to potential stress in humans. Neurosci Biobehav Rev.

[25] Seery MD, Weisbuch M, Blascovich J (2009). Something to gain, something to lose: the cardiovascular consequences of outcome framing. Int J Psychophysiol.

[26] Seery MD (2013). The biopsychosocial model of challenge and threat: using the heart to measure the mind. Soc Personal Psychol Compass.

[27] Moore LJ, Wilson MR, Vine SJ, Coussens AH, Freeman P (2013). Champ or chump?: challenge and threat states during pressurized competition. J Sport Exerc Psychol.

[28] Turner MJ, Jones MV, Sheffield D, Cross SL (2012). Cardiovascular indices of challenge and threat states predict competitive performance. Int J Psychophysiol.

[29] Blascovich J. In: Gardner JYSWL, editor. Challenge, threat, and health. New York, NY: Handbook of motivation science. Guilford Press; 2008. p. 481–93.

[30] Jefferson AL, Himali JJ, Beiser AS, Au R, Massaro JM, Seshadri S, Gona P, Salton CJ, DeCarli C, O'Donnell CJ, Benjamin EJ, Wolf PA, Manning WJ (2010). Cardiac index is associated with brain aging: the Framingham heart study. Circulation..

[31] O'Donovan A, Tomiyama AJ, Lin J, Puterman E, Adler NE, Kemeny M, Wolkowitz OM, Blackburn EH, Epel ES (2012). Stress appraisals and cellular aging: a key role for anticipatory threat in the relationship between psychological stress and telomere length. Brain behav Immun.

[32] Chalabaev A, Major B, Cury F, Sarrazin P (2009). Physiological markers of challenge and threat mediate the effects of performance-based goals on performance. J Exp Soc Psychol.

[33] Seery MD, Weisbuch M, Hetenyi MA, Blascovich J (2010). Cardiovascular measures independently predict performance in a university course. Psychophysiology.

[34] Behnke M, Kaczmarek LD (2018). Successful performance and cardiovascular markers of challenge and threat: a meta-analysis. Int J Psychophysiol.

[35] Hase A, O'Brien J, Moore LJ, Freeman P (2019). The relationship between challenge and threat states and performance: a systematic review. Sport Exerc Perform Psychol.

[36] LeDoux JE (1996). The emotional brain: the mysterious underpinnings of emotional life.

[37] Nisbett RE, Wilson TD (1977). Telling more than we can know - verbal reports on mental processes. Psychol Rev.

[38] Trotman GP, Williams SE, Quinton ML, van Zanten JJCS V (2018). Challenge and threat states: examining cardiovascular, cognitive and affective responses to two distinct laboratory stress tasks. Int J Psychophysiol.

[39] Turner MJ, Jones MV, Sheffield D, Slater MJ, Barker JB, Bell JJ (2013). Who thrives under pressure? Predicting the performance of elite academy cricketers using the cardiovascular indicators of challenge and threat states. J Sport Exerc Psychol.

[40] Quigley KS, Barrett LF, Weinstein S (2002). Cardiovascular patterns associated with threat and challenge appraisals: a within-subjects analysis. Psychophysiology.

[41] Braden AM, Osborne MS, Wilson SJ (2015). Psychological intervention reduces self-reported performance anxiety in high school music students. Front Psychol.

[42] Clark T, Williamon A (2011). Evaluation of a mental skills training program for musicians. J Appl Sport Psychol.

[43] Spahn C, Walther J-C, Nusseck M (2016). The effectiveness of a multimodal concept of audition training for music students in coping with music performance anxiety. Psychol Music.

[44] Hewitt MP (2015). Self-efficacy, self-evaluation, and music performance of secondary-level band students. J Res Music Educ.

[45] Williamon A, Valentine E (2000). Quantity and quality of musical practice as predictors of performance quality. Br J Psychol.

[46] Kubzansky LD, Stewart AJ (1999). At the intersection of anxiety, gender, and performance. J Soc Clin Psychol.

[47] McPherson GE, Schubert E, Williamon A (2004). Measuring performance enhancement in music. Musical excellence: strategies and techniques to enhance performance.

[48] Spitzer RL, Kroenke K, Williams JB (1999). Patient health questionnaire primary care study group. Validation and utility of a self-report version of PRIME-MD: the PHQ primary care study. Jama.

[49] Carballeira Y, Dumont P, Borgacci S (2007). Criterion validity of the French version of patient health questionnaire (PHQ) in a hospital department of internal medicine. Psychol Psychother-Theory Res Pract.

[50] Spielberger CD (1983). STAI state-trait anxiety inventory for adults form Y: review set; manual, test, scoring key.

[51] Spielberger CD, Bruchon-Schweitzer M, Paulhan I (1993). Inventaire d’Anxiété Etat-trait Forme Y (STAI-Y) [state-trait anxiety inventory, form Y].

[52] Cox WJ, Kenardy J (1993). Performance anxiety, social phobia, and setting effects in instrumental music students. J Anxiety Disord.

[53] Widmer S, Conway A, Cohen S, Davies P (1997). Hyperventilation: a correlate and predictor of debilitating performance anxiety in musicians. Med Probl Perform Artists.

[54] Kim Y (2005). Combined treatment of improvisation and desensitization to alleviate music performance anxiety in female college pianists: a pilot study. Med Probl Perform Artists.

[55] Kokotsaki D, Davidson JW (2003). Investigating musical performance anxiety among music college singing students: a quantitative analysis. Music Educ Res.

[56] Kenny DT (2011). The psychology of music performance anxiety.

[57] Kenny, DT. Kenny Music Performance Anxiety Inventory. 2017; Certified French translation.

[58] Kenny DT, Davis P, Oates J (2004). Music performance anxiety and occupational stress amongst opera chorus artists and their relationship with state and trait anxiety and perfectionism. J Anxiety Disord.

[59] Berntson GG, Bigger JT, Eckberg DL, Grossman P, Kaufmann PG, Malik M, Nagaraja HN, Porges SW, Saul JP, Stone PH, VanderMolen MW (1997). Heart rate variability: origins, methods, and interpretive caveats. Psychophysiology.

[60] Shapiro D, Jamner LD, Lane JD, Light KC, Myrtek M, Sawada Y, Steptoe A (1996). Blood pressure publication guidelines. Psychophysiology.

[61] Sherwood A, Allen MT, Fahrenberg J, Kelsey RM, Lovallo WR, Vandoornen LJP (1990). Methodological guidelines for impedance cardiography. Psychophysiology.

[62] Sackner MA, Watson H, Belsito AS, Feinerman D, Suarez M, Gonzalez G, Krieger B (1989). Calibration of respiratory inductive plethysmograph during natural breathing. J Appl Physiol.

[63] Moore LJ, Freeman P, Hase A, Solomon-Moore E, Arnold R. How consistent are challenge and threat evaluations? A generalizability analysis. Front Psychol. 2019;10:1778. 10.3389/fpsyg.2019.01778 PMC6687869 31428027

[64] Tomaka J, Blascovich J, Kelsey RM, Leitten CL (1993). Subjective, physiological, and behavioral effects of threat and challenge appraisal. J Pers Soc Psychol.

[65] Marteau TM, Bekker H (1992). The development of a six-item short-form of the state scale of the Spielberger state-trait anxiety inventory (STAI). Br J Clin Psychol.

[66] Cox RH, Martens MP, Russell WD (2003). Measuring anxiety in athletics: the revised competitive state anxiety inventory–2. J Sport Exerc Psychol.

[67] Martinent G, Ferrand C, Guillet E, Gautheur S (2010). Validation of the French version of the competitive state anxiety Inventory-2 revised (CSAI-2R) including frequency and direction scales. Psychol Sport Exerc.

[68] Yoshie M, Shigemasu K, Kudo K, Ohtsuki T (2009). Effects of state anxiety on music performance: relationship between the revised competitive state anxiety Inventory-2 subscales and piano performance. Music Sci.

[69] Lang PJ, Bradley MM, Cuthbert BN. International affective picture system (IAPS): Digitized photographs, instruction manual and affective ratings. Technical Report A-6Gainesville (FL): The Center for Research in Psychophysiology, University of Florida. 2005.

[70] Gil S (2009). Comment étudier les émotions en laboratoire ?. Rev Électronique Psychol Soc.

[71] Jackson SA, Eklund RC. Assessing flow in physical activity: the flow state scale-2 and dispositional flow scale-2. J Sport Exerc Psychol. 2002;24(2):133–50.

[72] Fournier J, Gaudreau P, Demontrond-Behr P, Visioli J, Forest J, Jackson S (2007). French translation of the flow state Scale-2: factor structure, cross-cultural invariance, and associations with goal attainment. Psychol Sport Exerc.

[73] Abbott MJ, Rapee RM. Post-event rumination and negative self-appraisal in social phobia before and after treatment. J Abnorm Psychol. 2004;113(1):136–44. 10.1037/0021-843X.113.1.136 14992666

[74] Spielberger CD (1989). State-trait anxiety inventory: bibliography.

[75] Fresco DM, Coles ME, Heimberg RG, Liebowitz MR, Hami S, Stein MB, Goetz D (2001). The Liebowitz social anxiety scale: a comparison of the psychometric properties of self-report and clinician-administered formats. Psychol Med.

[76] Liebowitz MR (1987). Social phobia. Mod Probl Pharmacopsychiatry.

[77] Yao SN, Note I, Fanget F, Albuisson E, Bouvard M, Jalenques I, Cottraux J (1999). Social anxiety in patients with social phobia: validation of the Liebowitz social anxiety scale: the French version. L'encéphale..

[78] Baker SL, Heinrichs N, Kim HJ, Hofmann SG (2002). The Liebowitz social anxiety scale as a self-report instrument: a preliminary psychometric analysis. Behav Res Ther.

[79] Beck AT, Steer RA, Ball R, Ranieri WF (1996). Comparison of Beck depression inventories-IA and -II in psychiatric outpatients. J Pers Assess.

[80] Bourque P, Beaudette D. Étude psychometrique du questionnaire de dépression de Beck auprès d'un échantillon d'étudiants universitaires francophones. Can J Behav Sci/Rev Can Sci Comportement. 1982;14(3):211–18.

[81] Pruessner JC, Kirschbaum C, Meinlschmid G, Hellhammer DH (2003). Two formulas for computation of the area under the curve represent measures of total hormone concentration versus time-dependent change. Psychoneuroendocrinology.

[82] Page-Gould E, Berntson GG, Cacioppo JT, Tassinary LG (2016). Multilevel modeling. Handbook of psychophysiology.

[83] West BT, Welch KB, Galecki AT (2015). Linear mixed models: a practical guide using statistical software.

[84] Baron RM, Kenny DA. The moderator–mediator variable distinction in social psychological research: conceptual, strategic, and statistical considerations. J Pers Soc Psychol. 1986;51(6):1173–82. 10.1037//0022-3514.51.6.1173 3806354

[85] Moore LJ, Vine SJ, Wilson MR, Freeman P (2012). The effect of challenge and threat states on performance: an examination of potential mechanisms. Psychophysiology..

[86] Cohen J (1988). Statistical power analysis for the behavioral sciences.

[87] Cleary PD, Barnett RS, Biener L, Baruch GK (1997). Gender differences in stress-related disorders. Gender and stress.

[88] Wittchen HU, Jacobi F, Rehm J, Gustavsson A, Svensson M, Jonsson B, Olesen J, Allgulander C, Alonso J, Faravelli C, Fratiglioni L, Jennum P, Lieb R, Maercker A, van Os J, Preisig M, Salvador-Carulla L, Simon R, Steinhausen HC (2010). The size and burden of mental disorders and other disorders of the brain in Europe. Eur Neuropsychopharmacol.

[89] Kudielka BM, Kirschbaum C (2005). Sex differences in HPA axis responses to stress: a review. Biol Psychol.

[90] Behan M, Wenninger JM (2008). Sex steroidal hormones and respiratory control. Respir Physiol Neurobiol.

[91] England SJ, Farhi LE (1976). Fluctuations in alveolar CO2 and in base excess during the menstrual cycle. Respir Physiol.

[92] Hamstra DA, de Kloet ER, Tollenaar M, Verkuil B, Manai M, Putman P, Van der Does W (2016). Mineralocorticoid receptor haplotype moderates the effects of oral contraceptives and menstrual cycle on emotional information processing. J Psychopharmacol.

[93] Kirschbaum C, Kudielka BM, Gaab J, Schommer NC, Hellhammer DH (1999). Impact of gender, menstrual cycle phase, and oral contraceptives on the activity of the hypothalamus-pituitary-adrenal axis. Psychosom Med.

[94] Bosch JA, de Geus EJ, Carroll D, Goedhart AD, Anane LA, van Zanten JJ, Helmerhorst EJ, Edwards KM (2009). A general enhancement of autonomic and cortisol responses during social evaluative threat. Psychosom Med.

