# Supplementary material for: Music performance anxiety from the challenge and threat perspective: psychophysiological and performance outcomes
Source: BMC Psychol. 2020 Aug 25;8:87. doi: 10.1186/s40359-020-00448-8 (PMC7448432; doi:10.1186/s40359-020-00448-8)
Supplement: Supplementary file 1 — Additional file 1. Music performance Quality (MPQ) Scale. This scale is used to evaluate the quality of the music performances. [file 40359_2020_448_MOESM1_ESM.docx]

**Music Performance Quality (MPQ) Scale**

We will ask you to evaluate the quality of the musical performance you just performed. To do this, a number of dimensions will be presented to you and you must indicate on a scale from 1 (= minimum score) to 6 (= maximum score) the quality of your performance on this dimension.

To help you in this task, information is provided for each dimension.

Please read these instructions carefully and do not hesitate to refer to them in case of doubt during your evaluation.

**Tempo**

Tempo refers to the execution speed of a piece of music (e.g., largo, adagio, andante, allegro) and to the time management of bars and/or tone groups. This may also include tempo variations (ex. accelerando, ritardando, unequal notes).

Please use the scale below (1 = worst mark, 6 = best mark) to evaluate the interpretation of the tempo of your performance in terms of stability, accuracy and consistency with the printed tempo markings and with the composer´s style.


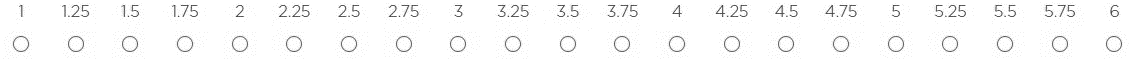


**Rhythm**

Rhythm refers to the musical event organization in time forming a temporal structure.

Please use the scale below (1 = worst mark, 6 = best mark) to evaluate the interpretation of the rhythmic patterns and characteristics of your performance in terms of accuracy, clarity, character and consistency with the composer´s style.


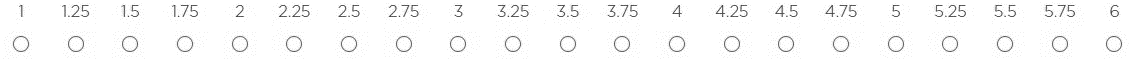


**Intonation**

Intonation refers to the accuracy of pitches in relation to each other and/or to a fixed standard.


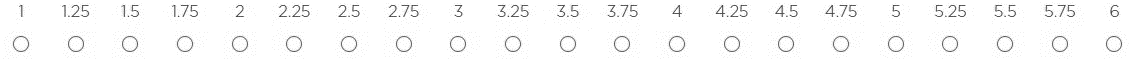
Please use the scale below (1 = worst mark, 6 = best mark) to evaluate the intonation quality of your performance in terms of accuracy and consistency across ranges and registers.

**Tone**

Tone refers to the sound produced by the instrument/the voice.

Please use the scale below (1 = worst mark, 6 = best mark) to evaluate the overall tone quality of your performance by referring to the projection, the color, the character, the variability and the control of the sound across ranges and registers as well as the richness of the overtone spectrum.


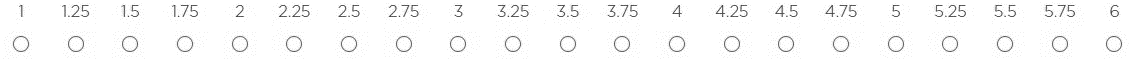


**Dynamics**

The dynamics of a piece refers to the variations in loudness between notes or phrases (e.g., piano, forte, crescendo, decrescendo, dynamic contrasts).


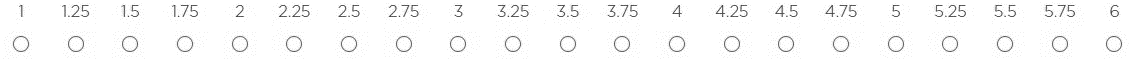
Please use the scale below (1 = worst mark, 6 = best mark) to evaluate the interpretation of the dynamics of your performance in terms of accuracy, expressivity, variability and consistency with the printed dynamics marking.

**Articulation**

Articulation refers to the way in which the transition from one sound to another occurs (e.g., legato, staccato, accents, ornamentation).


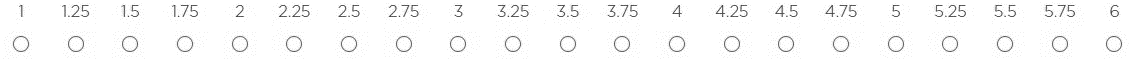
Please use the scale below (1 = worst mark, 6 = best mark) to evaluate the interpretation of the articulation of your performance in terms of accuracy, clarity and consistency with the printed articulation marking and with the composer´s style.

**Musical understanding and interpretation**

Musical understanding and interpretation refer to the performer’s understanding and respect of the intention, the aesthetic and the musical idea given by the composer to the piece. It also includes the expressive involvement of the performer in the music.


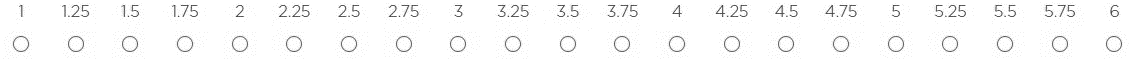
Please use the scale below (1 = worst mark, 6 = best mark) to evaluate the musical understanding and the interpretation of your performance.

**Missing notes, wrong notes and unwritten breaks**

Missing notes are notes which are written in the scores but which are omitted during music production.

Wrong notes are produced notes, which are different from the notes in the original score.

Unwritten breaks refer to pauses or stops not written in the original score.


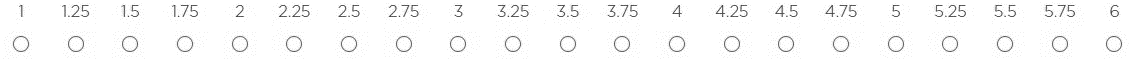
Please use the scale below (1 = A lot of missing, wrong notes and/or unwritten breaks, 6 = No missing, wrong notes and/or unwritten breaks) to evaluate globally the missing, wrong notes and/or unwritten breaks frequency in your performance.

**Global appreciation**

Global appreciation refers to the overall quality of the performance.


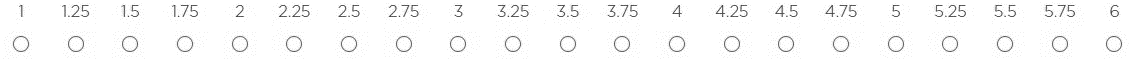
Please use the scale below (1 = worst mark, 6 = best mark) to evaluate the overall quality of your performance.

**Commentaries**
